# Health-related quality of life of rheumatic disease patients treated in a specialized IPS in Medellin, Colombia

**Published:** 2017

**Authors:** JQ Franco-Aguirre, AA Cardona-Tapias, JA Cardona-Arias

**Affiliations:** * Research Group on Health and Sustainability, School of Microbiology, University of Antioquia, Medellin, Colombia; ** School of Medicine, Cooperative University of Colombia; *** School of Microbiology, University of Antioquia U de A, Medellin, Colombia; School of Medicine,Cooperative University of Colombia

**Keywords:** quality of life, health care delivery, rheumatology, rheumatoid arthritis, fibromyalgia, spondyloarthritis

## Abstract

Purpose. To compare Health-Related Quality of Life (HRQOL) profiles and their associated factors in people with rheumatoid arthritis (RA), spondyloarthritis (SpA), fibromyalgia (FM) and rheumatoid comorbidity treated in a specialized health center (SHC) in Medellin, Colombia.

Methods. A cross-sectional analytical study was performed with 93 RA patients, 30 SpA patients, 41 primary FM patients and 48 secondary FM patients with a concurrent diagnosis of RA or SpA. A demographic, socioeconomic, and clinical survey (the IPAQ, International Physical Activity Questionnaire) and the SF-36 survey validated in Medellin were administered. The statistical analyses were executed using SPSS 21.0

Results. Significant differences were found in all HRQOL dimensions among the patients, with physical performance perceived as the worst in the four groups. FM had the worst HRQOL profile, whereas the least affected group was SpA. Patients with RA and rheumatoid comorbidity shared similar HRQOL scores. The years of study, age and economic satisfaction variables were associated with the physical performance, vitality, social functioning, and mental health domains.

Conclusion. The HRQOL profile was negative in patients with rheumatic diseases and lower in patients with FM. Additionally, variables or subgroups with greater deterioration were identified. This information will be useful for health activities and generate evidence in favor of incorporating HRQOL measurements into rheumatology services to complement clinical evaluations.

## Background

Rheumatic diseases constitute a group of chronic conditions that involve the musculoskeletal system. The individual and social impacts of these diseases are associated with a decreased quality of life for patients and their families, a loss of productivity and increased health service costs. Additionally, a rising life expectancy is one of the main factors associated with increased diagnosis [**1**].

The World Health Organization (WHO) declared the years 2000-2010 the "Bone and Joint Decade" to emphasize the lack of attention given to bone and joint diseases by health professionals, the importance of improving the quality of life of people with musculoskeletal conditions and the need for their inclusion in public health policy. This attention is necessary because these diseases are generally regarded as a natural part of aging and do not represent a major threat to human life [**1**][**2**].

In this group of diseases, rheumatoid arthritis (RA), fibromyalgia (FM) and spondyloarthritis (SpA) are of great importance because of their prevalence, complex etiology and wide geographical distribution [**1**][**3**]-[**5**]. RA is a progressive, inflammatory, and chronic autoimmune disease that primarily involves the joints. The clinical manifestations of RA include swelling and joint pain, morning stiffness, fatigue and reduced mobility; its prevalence is estimated to be between 0.5% and 1.0% of the adult population, with a higher proportion of women affected [**1**][**4**][**6**].

FM represents a disease of unknown etiology characterized by chronic and diffuse musculoskeletal pain in different anatomical sites (primarily in the non-axial skeleton). The symptoms of FM include fatigue, sleep disorders, morning stiffness, a swelling sensation in the hands, headaches, irritable bowel syndrome, anxiety and depression; its estimated prevalence varies between 0.7% and 4.4% [**3**][**7**].

The SpAs (ankylosing spondylitis, reactive arthritis, psoriatic arthritis, a subgroup of juvenile chronic arthritis and undifferentiated spondyloarthritis) are a group of diseases whose global prevalence has been estimated to be between 0.1% and 1.6%. From a clinical perspective, they share the same pattern of manifestations (i.e., the involvement of the peripheral joints, especially the lower limbs, and the possible occurrence of sacroiliitis, enthesitis, and uveitis). Affected patients complain of severe pain and stiffness with a consequent reduction in mobility and physical functioning [**5**][**8**].

Traditionally, the study of these diseases has focused on the assessment of biochemical and radiological parameters, functioning of the musculoskeletal system and disability grading measurements. However, this clinical approach does not consider other important domains in an individual’s daily life that are negatively impacted by the disease [**9**][**11**]. Moreover, these clinical parameters are insufficient to assess the quality of health services and decision-making. Therefore, there is a need for complementary indicators that demonstrate the impact of the diseases on patient quality of life [**9**][**11**].

The scientific literature provides numerous studies related to research, on the HRQOL of patients with rheumatic diseases. These studies are evidenced by the systematic reviews of Franco, Cardona, and Hernandez [**12**][**14**], who have described different studies from around the world regarding the HRQOL of individuals with psoriasis, RA and FM over the past decade.

Among the most important findings of these reviews, the identification of the main objectives in each of the studies characterized highlights: i) determining the HRQOL profile; ii) evaluating the HRQOL as a secondary outcome to an intervention; iii) validating an instrument. These reviews also determined that the Medical Outcome Study Short Form-36 (MOSSF-36) was the most commonly used health survey among the different HRQOL instruments used in rheumatology. This preference is due to the excellent validity and reliability of the MOSSF-36 combined with its good correlation with specific clinical measurements [**15**][**24**]. Finally, based on the key findings of these reviews, it is worth noting the high concentration of studies in Europe and North America and the low proportion of HRQOL research based in Colombia (four studies on FM and seven on RA). Moreover, there is an absence of comparative studies of the HRQOL profiles across these diseases.

Background research comparing the HRQOL profiles among rheumatic diseases includes the study of Salaffi et al. [**25**] in Italy that investigated patients with RA, ankylosing spondylitis (AS) and psoriatic arthritis. Their results showed a greater involvement of the physical component domains in individuals with RA. Borman et al. [**26**] found a greater involvement of the physical disability domains and pain in patients with RA compared with patients affected by psoriatic arthritis in Turkey. Tander et al. [**27**] and Ovayolu et al. [**28**] conducted comparative studies of patients with RA and FM and patients with FM, AR and AS in Turkey, respectively. These authors found lower scores in the HRQOL domains among individuals with FM (especially the domains related to the mental component). All these studies identified factors associated with the HRQOL in addition to the disease, including various clinical and socio-demographic characteristics such as body mass index, disease duration, age, education level, income, and occupational status.

Conducting a comparative study of the HRQOL among individuals with RA, FM, or SpA is of great importance, because it permits the identification of the HRQOL dimensions that are most affected by each disease. Moreover, the HRQOL assessment allows a comparison of the degree of involvement or the impact of each diagnosis as a basis for prioritizing the care, prevention or other activities of patients attending rheumatology services. Additionally, this approach allows the implementation of public health policies aimed at improving the quality of life in the affected individual.

In line with the above points, the objectives of this study were: evaluating and comparing the HRQOL profiles across people with rheumatoid arthritis (RA), spondyloarthritis (SpA), fibromyalgia (FM) and rheumatoid comorbidity treated in a specialized health center (IPS) in Medellin-Colombia and identifying the associated factors to the HRQOL.

## Methods

**Type of study**

Cross-sectional analytical study.

**Site**

The institution providing health services is a specialized center for rheumatology consultation serving approximately 500 patients per month from most of the metropolitan area of the Aburra Valley in Colombia.

**Research subjects**

Inclusion criteria: individuals diagnosed with RA according to the 2010 classification criteria of the ACR (American College of Rheumatology)/ EULAR (European League Against Rheumatism) [**29**], individuals diagnosed with some type of SpA according to the 1991 classification criteria of the European Spondyloarthropathy Study Group [**30**], individuals diagnosed with fibromyalgia according to the 2010 ACR criteria [**31**], and patients with a concurrent diagnosis of FM and RA or SpA. All subjects were 18 years or older, had a consultation with the rheumatologist and were selected using a non-probabilistic method.

Exclusion criteria: individuals who could generate an information bias by cognitive problems or drugs abuse according to medical criteria were excluded, joined by patients who refused to participate in the study.

**Data Collection**

A survey was designed to obtain demographic, socio-economic and clinical information such as sex, age, marital status, socioeconomic status, health system affiliation, satisfaction with family support and economic status, participation in social groups, body mass index (BMI), alcohol and/ or tobacco use, presence of comorbidities, hospitalizations in the past year and illnesses or accidents in the last month; the IPAQ scale (International Physical Activity Questionnaire) [**32**] was implemented to assess physical activity and sedentary lifestyle. The evaluation of the health-related quality of life was made through the generic questionnaire: MOSSF-36 [**34**].

Data collection from secondary sources (clinical history) to obtain measurements of RA and SpA inflammatory activity proved ineffective due to gaps in the timing of the clinometric measurements and the evaluation of the HRQOL greater than 1 month, which would represent a skewed reality of the HRQOL associated with no recent clinical status.

**Validated measurement scales**

The IPAQ short version consists of five questions concerning the frequency, duration, and intensity (moderate and vigorous) of physical activity performed in the last 7 days, as well as walking and sedentary time on a working day. It permits the assignment of individuals to three categories of physical activity: low (sedentary), moderate or high. Individuals in the low category do not meet the criteria for the moderate or high categories [**32**]. This questionnaire was culturally adapted for Colombia based on the IPAQ employed in the Hispanic population in the United States in national nutrition surveys between 2005 and 2010 [**33**].

The SF-36 health survey is a valid instrument for the assessment of the health status and HRQOL in healthy and sick people. It consists of a survey of 36 questions that generates a profile with 8 dimensions: body pain, physical performance, physical functioning, general health (physical component summary), emotional function, social function, mental health and vitality (mental component summary). The score of each one of the dimensions can range from 0 (worst state) to 100 (best state) with a reference value for the physical and mental components established in a healthy population of 50 ± 10 [**34**]. Among its psychometric properties, we highlight its reliability based on a Cronbach's α value higher than 0.7 [**35**][**36**], applied to a healthy population and different groups of patients (diabetes, depression and musculoskeletal disease) assessed in Medellin (Colombia) [**37**]. Moreover, we established reference values for this population using the comprehensive study of Garcia et al. in 2013 [**38**].

**Control of bias**

Selection bias was controlled by the rigorous application of inclusion criteria that guaranteed the adequate selection of research subjects. The study used the recommended criteria validated by rheumatology associations with international recognition (ACR and EULAR).

Measurement bias in critical variables such as the HRQOL and physical activity was controlled by applying instruments with satisfactory results, which were well-documented worldwide regarding their psychometric properties of validity and reliability. The biases of the observers were controlled by training on issues related to data collection protocols and ethical and technical aspects of the research project. An assessment of their suitability and cognitive ability to participate in the study was conducted by specialized IPS medical staff to control for bias attributable to the research subjects.

**Data analysis plan**

A description of the socio-demographic and clinical characteristics of the four research groups was provided based on proportions and summary measurements analyzed with the Chi-squared and Kruskal-Wallis H tests. These tests were also used to evaluate significant differences between groups and their characteristics. 

The scores of the HRQOL dimensions were compared across the study groups with the Kruskal Wallis H test because the data were not normally distributed and evaluated with the Shapiro-Wilk and Kolmogorov-Smirnov with Lilliefors correction. In the dimensions that showed an association with the group, an analysis of multiple comparisons was performed using the Mann-Whitney U test with Bonferroni correction. The association of demographic, socioeconomic, and clinical variables with the HRQOL dimensions of the SF-36 was assessed using the Mann Whitney U test and Spearman's rank correlation coefficient. Because some independent variables revealed a significantly different distribution among the four study groups, three conditions were used to evaluate potential confounding factors: i) the factor was not an intermediate step in the causal event horizon; ii) the variable might reveal an association with the study group or illness; iii) the variable might reveal an association with one or more dimensions of quality of life. Therefore, the quantification of confounding factors was performed with multiple linear regression models prior to verifying the assumptions of randomness of the dependent variables (each dimension of the HRQOL) with the Runs test, the linearity in ANOVA, the normality and constant variance of the residuals, the autocorrelation in residuals using the Durbin Watson test, the lack of collinearity between independent variables through the variance inflation factor (VIF) and the statistical significance of the regression coefficients.

The analyses were performed with a significance level of 0.05 in SPSS 21.0

**Ethical aspects**

The project complied with the ethical guidelines of the Declaration of Helsinki and Resolution 8430 of the Ministry of Health of Colombia and was supported by the Bioethics Committee of the Cooperative University of Colombia Code 0800-0034.

## Results

HRQOL was evaluated and compared in a total of 212 patients in four study groups composed of 93 diagnosed with AR, 30 diagnosed with SpA, 41 FM and 48 patients with rheumatic comorbidity (AR/ SpA + FM). Two people were excluded based on medical criteria that could generate biased information on cognitive impairments or the use of hallucinogens, and 7 patients refused to participate in the study.

In the comparison of the demographic, social, economic, and clinical characteristics among the four research groups, significant differences were found for the variables sex, age, monthly income, health membership, satisfaction with the economic situation, social, or community participation, presence of comorbidities and hospitalization in the last year (**Table 1**).

**Table 1:** Percentage distributions of clinical and socio-demographic characteristics of the study population

Dichotomous Variables	FM, N=41 (%)	RA /SpA + FM, N=48 (%)	RM, N=93 (%)	SpA, N=30 (%)	Pv Chi^2^
Sex (Women)	95.1	87.5	86.0	30.0	0.000**
Civil Status (Married)	63.4	56.3	54.8	66.7	0.605
Social Stratum (Medium-High)	63.4	45.8	51.6	63.3	0.260
Health Affiliation (Contributory)	92.7	91.7	77.4	90.0	0.037*
Family support satisfaction	85.4	68.8	71.0	76.7	0.261
Economic situation satisfaction	73.2	52.1	25.8	20.0	0.000**
Social participation	34.1	27.1	14.0	3.3	0.003**
Sedentary	68.3	70.8	63.4	60.0	0.724
Alcohol Consumption	17.1	6.2	10.8	16.7	0.348
Smoking	9.8	16.7	9.7	13.3	0.631
Comorbidity	68.3	100.0	69.9	63.3	0.000**
Hospitalization	29.3	43.8	62.4	53.3	0.003**
Polytomous variables					
Occupation					
Employed	41.5	33.3	34.4	56.7	0.197
House work	46.3	37.5	46	20.0	
Other activities	4.9	20.8	14.0	16.7	
Disabled	7.3	8.3	5.4	6.7	
BMI category					
Normal	48.8	41.7	54.8	40.0	0.372
Overweight	29.3	33.3	33.3	43.3	
Obese	22.0	25.0	11.8	16.7	
Quantitative variables	Median (interquartile range)				Pv KW^‡^
Age	51 (46-62)	53 (44-61)	54 (48-60)	49 (37-54)	0.042*
Monthly income (US)	236 (0-236)	237 (0-480)	215 (0-334)	258 (208-626)	0.004***
Years of study	9 (5-11)	11 (5-12)	11 (5-11)	11 (5-13)	0.051
*Pvlt;0.05. **Pv<0.01. ‡P-values for Kruskall Wallis test. 1 US$ 2015 = 3000 COP.					

Women were the predominant sex in all groups; the exception was the SpA group. Approximately half of the patients in the four groups belonged to the low socio-economic strata, the married civil status was the most prevalent, and 50% of the central values of age and schooling corresponded to young adults and persons with a basic secondary education (**Table 1**).

In the four groups, a high frequency of participants who were overweight or obese (greater than 45%) reported physical inactivity, comorbidities and hospitalization within the last year (**Table 1**).

The best score in the HRQOL profiles of the four study groups corresponded to the dimension of general health among patients with fibromyalgia, whereas the worst score was among physical performance. In the rheumatoid arthritis group, the highest score was in social functioning and the lowest was in physical performance. In the spondyloarthritis group, the highest average was observed in mental health and the lowest was in physical performance (**Table 2**).

**Table 2:** Comparison of HRQOL profiles among the study groups

	Fibromyalgia		FM + RA + SpA		Rheumatoid Arthritis		Spondyloarthropathies		Pv
	X^±DE^	Me (RI)	X^±SD^	Me (RI)	X^±SD^	Me (RI)	X^±SD^	Me (IR)	K-W^‡^
PF	33^ ±20^	30 (20-40)	46^ ±25^	43 (30-62)	48^ ±26^	45 (30-65)	62^ ±32^	70 (35-90)	0,000
PP	12^ ±26^	0 (0-0)	26^ ±39^	0 (0-50)	34^ ±43^	0 (0-100)	44^ ±47^	25 (0-100)	0,012
BP	23^ ±22^	22 (0-44)	40^ ±26^	44 (22-56)	46^ ±23^	44 (33-56)	56^ ±34^	56 (33-89)	0,000
GH	38^ ±21^	38 (21-50)	43^ ±16^	43 (35-50)	40^ ±12^	40 (35-45)	49^ ±22^	45 (35-65)	0,038
VT	31^ ±19^	25 (20-40)	44^ ±26^	40 (25-63)	46^ ±27^	40 (25-70)	57^ ±26^	60 (45-80)	0,000
SF	47^ ±36^	25 (25-88)	53^ ±29^	50 (31-75)	71^ ±27^	75 (50-100)	63^ ±37^	75 (25-100)	0,000
EP	22^ ±35^	0 (0-33)	45^ ±48^	17 (0-100)	58^ ±49^	100 (0-100)	64^ ±48^	100 (0-100)	0,000
MH	43^ ±20^	36 (28-56)	57^ ±24^	52 (40-76)	63^ ±23^	64 (48-80)	71^ ±25^	74 (56-92)	0,000
X: Mean. ST: Standard Deviation. Me: Median. IR: Interquartile Range, ‡ P values for Kruskall Wallis test. PF: Physical Functioning. PP: Physical Performance. BP: Body Pain. GH: General Health. VT: Vitality. SF: Social Functioning. EP: Emotional Performance. MH: Mental Health.									

A comparison of the HRQOL in the four groups showed significant differences in all dimensions of the SF-36 health survey. In the multiple comparisons, the following results were revealed:

1. Fibromyalgia patients showed significantly lower scores compared with the rheumatoid arthritis group in all dimensions except with general health, which was statistically equal.

2. In the comparison between fibromyalgia and spondyloarthropathy, all dimensions revealed poorer scores in the first group with the exception of social functioning, which did not differ between the groups.

3. Only differences in overall health and vitality were found among the patients with rheumatoid arthritis and spondyloarthropathy.

4. The group with fibromyalgia was significantly different from the group that represented rheumatic comorbidity in the physical functioning, body pain, vitality, mental health, and emotional performance dimensions.

5. In the group that represented rheumatic comorbidity, differences were found compared with rheumatoid arthritis in social functioning and compared with spondyloarthropathy for the dimensions physical functioning, body pain, vitality, and mental health.

Physical performance was perceived as the worst domain among the four groups, whereas the body pain, mental health and emotional performance domains in patients with fibromyalgia were significantly more affected compared with the patients in the remaining groups. In general, fibromyalgia was the disease with the worst HRQOL profile, whereas spondyloarthritis had the least affected HRQOL profile. The HRQOL scores of patients with RA were similar to those with rheumatic comorbidities (**Table 2**).

In terms of the independent variables associated with the HRQOL, sex was associated with vitality (Pv = 0.034) and physical functioning (Pv = 0.029), economic satisfaction was associated with vitality (Pv = 0.042), and hospitalization within the past year was associated with physical functioning (Pv = 0.010), physical performance (Pv = 0.016), body pain (Pv = 0.023) and emotional performance (Pv = 0.044). For the quantitative variables, age was weakly correlated with social functioning (Spearman Rho = 0.137), years of study was weakly correlated with the domains physical functioning (Spearman Rho = 0.188), physical performance (Spearman Rho = 0.244), vitality (Rho Spearman = 0.175), social functioning (Spearman Rho = 0.148), emotional performance (Spearman Rho = 0.137) and mental health (Spearman Rho = 0.231), and income was weakly correlated with physical functioning (Spearman Rho = 0.198) physical performance (Spearman Rho = 0.197), body pain (Spearman Rho = 0.140), emotional performance (Spearman Rho = 0.172) and mental health (Spearman Rho = 0.188).

These data highlighted the possibility that the significant differences in the HRQOL dimensions among the four study groups could be affected by the independent variables that showed associations with some HRQOL scores (i.e., the three conditions for a potentially confounding effect were met). This result demonstrated why the multivariate adjustments were performed: we sought to establish whether all of the significant differences found in the bivariate analysis were "real" or whether some were the result of an effect modification (confounding type). 

The data in **Table 3** revealed that the differences in the scores of the HRQOL dimensions among the four study groups were not affected by the other independent variables (i.e., the HRQOL profile was significantly different in people with fibromyalgia, rheumatoid arthritis and spondyloarthropathies following adjustment for independent variables such as sex, age, schooling, hospitalization and other services included in the regression model).

**Table 3:** Regression models for the adjustment of independent variables associated with the HRQOL

HRQOL Dimension	Variables of the Model	Regression Coefficient
Physical Functioning	Disease^ɬ^	6.274**
	Sex (Male/Female)	-3.666
	Hospitalization (No/Yes)	5.319
	Income	0.006
	Years of Study	0.908
Physical Performance	Disease^ɬ^	8.932**
	Years of Study	2.788**
	Hospitalization (No/Yes)	12.170*
	Income	-0.007
Body Pain	Disease^ɬ^	10.441**
	Hospitalization (No/Yes)	2.843
	Income	0.008
General Health	Disease^ɬ^	2.427*
Vitality	Disease^ɬ^	9.526**
	Economic Satisfaction	15.466**
	Years of School	1.002*
	Sex	-3.595
	Income	-0.003
Social functioning	Disease^ɬ^	7.916**
	Years of Study	1.518**
	Age	0.521**
Emotional Performance	Disease^ɬ^	13.071**
	Hospitalization (No/Yes)	8.333
	Years of Study	1.314
	Income	0.000
Mental Health	Disease^ɬ^	7.990**
	Years of Study	1.024*
	Income	0,002
*Pv < 0.05 **Pv < 0.01. ^ɬ^Order of Disease Categories: Fibromyalgia, FM + AR / SpA, rheumatoid arthritis, spondyloarthropathies.		

Furthermore, the year of study was identified as a variable associated with physical performance, vitality, social functioning, and mental health, and age was identified as a factor associated with social functioning.

The data indicated that each additional year of life represented 0.5 points in the final score of this domain. Hospitalization in the last year represented a significant decrease in the physical performance score (12.2 points), and satisfaction with the economic situation represented better scores in the vitality domain (15.5 points) compared to those individuals who reported that they were dissatisfied (**Table 3**).

## Discussions

The present study found significant differences in all HRQOL dimensions among the patients, with physical performance perceived as the worst in the four groups. FM had the worst HRQOL profile, whereas the least affected group was SpA. Patients with RA and rheumatic comorbidity shared similar HRQOL scores. The years of study, age and economic satisfaction variables were associated with the physical performance, vitality, social functioning, and mental health domains.

The broad spectrum of rheumatic diseases that have a negative impact on the HRQOL of individuals is evident. Among them, FM represents a condition in which the daily life of the subject is severely affected, even compared to other conditions of the same type such as RA and SpA [**40**], which comprise the conditions with the lowest impact on quality of life in the studied population.

In the study of chronic diseases, using the HRQOL, conducted in Holland by Sprangers et al. [**40**], diseases of the musculoskeletal system had a higher negative impact on the quality of life of an individual than cardiovascular, renal, and neurological diseases and cancer. Similarly, gross differences were evident when the HRQOL was compared across this group of diseases and healthy populations [**25**][**28**][**37**][**39**][**41**]. Thus, the mean scores obtained in each domains evaluated for the study population, showed significant clinical differences (over 5 points) compared to reference values obtained for the healthy population in the city of Medellin by Garcia et al. [39]: PF = 90,7±14,6 vs. 46,9±26,6, RP = 86,9 ± 27,5 vs. 29,1±40,9, BP = 80,8±21,5 vs. 41,2 ±26,9, GH = 78±18,1 vs. 41,6±16,9, VT = 73.5±16 vs. 44,2±26,1, SF = 82,8 ±20,2 vs. 60,9±32,2, RE = 83,8 ±30,8 vs. 49,1±48,2; MH = 75,7±17,1 vs. 58,7±24,2). Therefore, these types of conditions deserve special attention on the part of health decision makers, who should establish a longitudinal HRQOL assessment as a basic activity in disease monitoring and surveillance to achieve therapeutic goals.

The scores obtained in the HRQOL dimensions for individuals with FM revealed that this illness had the worst HRQOL profile. Similarly, Birtane et al. [**42**], Tander et al. [**27**], Ovayolu et al. [**28**] and Salaffi et al. [**41**] reported lower scores in some of the components or domains of the SF-36 (especially those related to mental health) in their comparative studies of FM with RA and ankylosing spondylitis.

In this sense, the HRQOL profile in subjects with the concurrent presence of two rheumatic diseases should be inferior compared with an individual who suffers only from FM. However, the results obtained in this study suggest otherwise because a stronger profile was identified for individuals with rheumatic comorbidity (FM + AR/ SpA).

This finding could be explained through the theory of the pathogenesis of type 2 FM proposed by Belenguer et al. [**43**], who state the appearance of this condition as a result of the pathophysiological mechanisms triggered by an underlying disease that corresponded to the majority of cases of RA and some cases of SpA. In this sense, a history of rheumatic disease assumes the activation of various coping mechanisms that can mitigate the negative perception of the HRQOL at the time of FM diagnosis.

In turn, among the factors associated with the HRQOL, years of study constituted a socio-demographic characteristic that might explain the HRQOL profile. Thus, people with higher education reported better scores in several domains of the SF-36 survey. These results were akin to the results reported by Salaffi et al. [**25**][**41**] and Ovayolu et al. [**28**] who identified the level of education as a mitigating factor of a poor HRQOL. This effect was attributable to the fact that a higher educational background allowed a better understanding of the disease, greater efficacy, and excellent control of disabling situations.

Economic satisfaction affected the perception of vitality in a positive way that was similar to the report by Ovayolu et al. [**28**], who indicated that precarious economic conditions were associated with a poor HRQOL. Alshiri et al. [**44**] found that income was a predictor of the HRQOL in patients with RA, and Cardona-Arias et al. [**18**] reported that this variable was a factor associated with physical functioning in patients with FM. These data show the importance of capacity and economic satisfaction as mitigating aspects of unfavorable conditions fostered by the disease (from increased availability of resources to the search for mechanisms to cope with the condition).

Finally, the association between age and social functioning explained that the increase in years of life in individuals improved the perception of this dimension of the HRQOL. Lopez-Garcia et al.’s [**45**] study of baseline SF-36 survey data of a population over 60 years of age attributed the low impact of age on the mental component domains to a sort of "survival effect" in which the individuals involved had already exceeded their life expectancy with a consequent improvement in the perception of emotional well-being regardless of the detriment of their physical well-being.

The limitations of the study include the unavailability of simultaneous clinimetric measurements of patients or measurements temporally aligned with the assessment of patient HRQOL, exploratory statistical associations and temporal bias in cross-sectional studies. However, the results allowed the formulation of hypotheses for further research related to factors associated with the HRQOL profile among patients in each group and the differences in the degree of involvement or impact by the type of diagnosis.

## Conclusions

The rheumatic diseases addressed in this study constituted conditions whose negative impacts were relevant in the daily lives of the affected individuals. Additionally, FM represented the condition with the highest perception of disability in the physical, emotional, and social dimensions, whereas RA, SpAs and rheumatic comorbidity showed less involvement across these dimensions.

The HRQOL evaluation constitutes an activity that should be prioritized to complement traditional clinical evaluations in rheumatology services because it allows the provider to focus on more effective and timely care models according to the profiles of the identified quality of life. Furthermore, the identification of factors associated with the HRQOL suggests that information on the demographic, socioeconomic, and clinical characteristics of the individual should be integrated into its assessment to ensure a better understanding and intervention of the affected dimensions and to achieve better adherence to biological, psychological, and social intervention protocols.

## Consent

A written informed consent was obtained from the patient for the publication of this case report and any accompanying images. A copy of the written consent is available for review by the Editor-in-Chief of this journal.

## Competing Interests

The authors declare that they have no competing interests.

## Funding

Resources in kind from the University of Antioquia, Sustainability Strategy, Cooperative University of Colombia, 2014.

## Authors' Contributions

JQFA: Acquisition, analysis and interpretation of data; drafting and revising the manuscript; final approval of the version to be published; agreement to be held accountable for all aspects of the work in ensuring that questions related to the accuracy or integrity of any part of the work are appropriately investigated and resolved.

AACT: Acquisition of data; drafting and revising the manuscript; final approval of the version to be published; agreement to be held accountable for all aspects of the work in ensuring that questions related to the accuracy or integrity of any part of the work are appropriately investigated and resolved.

JACA: Conception and design; acquisition, analysis and interpretation of data; drafting and revising the manuscript; final approval of the version to be published; agreement to be held accountable for all aspects of the work in ensuring that questions related to the accuracy or integrity of any part of the work are appropriately investigated and resolved.

## Authors' Information

JQFA: Teaching in University of Antioquia.

AACT: Rheumatologist and Teaching School of Medicine, University Cooperative of Colombia.

JACA: Teaching School of Microbiology, University of Antioquia; School of Medicine, University Cooperative of Colombia.

## Acknowledgements

To the patients.

## References

[1] Chopra A, Abdel-Nasser A (2008). Epidemiology of rheumatic musculoskeletal disorders in the developing world. Best Practice & Research Clinical Rheumatology.

[2] Woolf AD (2000). The bone and joint decade 2000–2010. Annals of the Rheumatic Diseases.

[3] Cavalcante A, Sauer J, Chalot S, Assumpção A, Lage L, Matsutani L (2006). Prevalência de Fibromialgia: uma Revisão de Literatura. Revista Brasileira de Reumatologia.

[4] Rat A, El Adssi H (2013). Epidemiology of rheumatic diseases. EMC-Aparato Locomotor.

[5] Sieper J, Rudwaleit M, Khan MA, Braun J (2006). Concepts and epidemiology of spondyloarthritis. Best Practice & Research Clinical Rheumatology.

[6] (c2011). Update of the clinical practice guide for the management of rheumatoid arthritis in Spain [Internet].

[7] Wierwille L, Abdel-Nasser A (2012). Fibromyalgia: diagnosing and managing a complex syndrome. Journal of the American Academy of Nurse Practitioners.

[8] Spondyloarthritis [Internet].

[9] Schwartzmann L (2003). Health related quality of life: conceptual aspects. Ciencia y Enfermería.

[10] Ruiz MA, Pardo A (2005). Health related quality of life: definition and use in medical practice. Pharmacoeconomics Spanish Research Articles.

[11] Madrigal M, Velandrino A, Ruzafa M Evaluation of studies related to health related quality of life [Internet].

[12] Franco-Aguirre  JQ, Cardona-Arias JA (2014). Characterization of studies on health-related quality of life in people with psoriasis: a systematic review 2003–2013. Revista Colombiana de Reumatología.

[13] Hernández-Petro AM, Cardona-Arias JA (2014). Systematization of research on health-related quality of life in fibromyalgia, 2004-2014. Archivos de Medicina.

[14] Franco J, Cardona J (2015). Health related quality of life to in people with rheumatoid arthritis: characterization of studies published between 2003-2013. Iatreia.

[15] Feroz AHM, Islam MN, Klooster PM, Hasan M, Rasker JJ, Haq SA (2012). The Bengali short Form-36 was acceptable, reliable, and valid in patients with rheumatoid arthritis. Journal of Clinical Epidemiology.

[16] Koh ET, Leong KP, Tsou IY, Lim VH, Pong LY, Chong SY (2006). The reliability, validity and sensitivity to change of the Chinese version of SF-36 in oriental patients with rheumatoid arthritis. Rheumatology (Oxford).

[17] Loge JH, Kaasa S, Hjermstad MJ, Kvien TK (1998). Translation and performance of the Norwegian SF-36 Health Survey in patients with rheumatoid arthritis. I. Data quality, scaling assumptions, reliability, and construct validity. Journal of Clinical Epidemiology.

[18] Cardona-Arias JA, Hernández-Petro AM, León-Mira V (2014). Validity, reliability and internal consistency of three measuring instruments of health-related quality of life in people with fibromyalgia, Colombia. Revista Colombiana de Reumatología.

[19] Leung YY, Ho KW, Zhu TY, Tam LS, Kun EW, Li EK (2010). Testing scaling assumptions, reliability and validity of medical outcomes study short-form 36 health survey in psoriatic arthritis. Rheumatology (Oxford).

[20] Birrell FN, Hassell AB, Jones PW, Dawes PT (2000). CHow does the short form 36 health questionnaire (SF-36) in rheumatoid arthritis (RA) relate to RA outcome measures and SF-36 population values? A cross-sectional study. Clinical Rheumatology.

[21] Kvien TK, Kaasa S, Smedstad LM (1998). Performance of the Norwegian SF-36 health survey in patients with rheumatoid arthritis. II. A comparison of the SF-36 with disease-specific measures. Journal of Clinical Epidemiology.

[22] Grozdev I, Kast D, Cao L, Carlson D, Pujari P, Schmotzer B (2012). Physical and mental impact of psoriasis severity as measured by the compact short form-12 Health Survey (SF-12) quality of life tool. Journal of Investigative Dermatology.

[23] Jajić Z, Rajnpreht I, Kovačić N, Lukić IK, Velagić V, Grubišić F (2012). Which clinical variables have the most significant correlation with quality of life evaluated by SF-36 survey in Croatian cohort of patient with ankylosing spondylitis and psoriatic arthritis?. Rheumatology International.

[24] Revicki DA, Rentz AM, Luo MP, Wong RL (2011). Psychometric characteristics of the short form 36 health survey and functional assessment of chronic illness therapy-fatigue subscale for patients with ankylosing spondylitis. Health and Quality of Life; Outcomes.

[25] Salaffi F, Carotti M, Gasparini S, Intorcia M, Grassi W (2009). The health-related quality of life in rheumatoid arthritis, ankylosing spondylitis, and psoriatic arthritis: a comparison with a selected sample of healthy people. Health and Quality of Life; Outcomes.

[26] Borman P, Toy GG, Babaoğlu S, Bodur H, Cılız D, Allı N (2007). A comparative evaluation of quality of life and life satisfaction in patients with psoriatic and rheumatoid arthritis. Clinical Rheumatology.

[27] Tander B, Cengiz K, Alayli G, İlhanli İ, Canbaz S, Canturk F (2008). A comparative evaluation of health related quality of life and depression in patients with fibromyalgia syndrome and rheumatoid arthritis. Rheumatology International.

[28] Ovayolu N, Ovayolu O, Karadag G (2011). -related quality of life in ankylosing spondylitis, fibromyalgia syndrome, and rheumatoid arthritis: a comparison with a selected sample of healthy individuals. Clinical Rheumatology.

[29] Aletaha D, Neogi T, Silman A, Funovits J, Felson D, Bingham C (2010). Rheumatoid arthritis classification criteria: an American College of Rheumatology/European League Against Rheumatism collaborative initiative. Arthritis & Rheumatism.

[30] Dougados Z, Linden SVD, Juhlin R, Huitfeldt B, Amor B, Calin A (1991). The European Spondylarthropathy Study Group preliminary criteria for the classification of spondylarthropathy. Arthritis & Rheumatism.

[31] Wolfe F, Clauw DJ, Fitzcharles M, Goldenberg DL, Katz RS, Mease P (2010). The American College of Rheumatology preliminary diagnostic criteria for fibromyalgia and measurement of symptom severity. Arthritis Care & Research.

[32] International Physical Activity Questionnaires [Internet].

[33] (c2010). National survey of nutritional situation in Colombia. ENSIN [Internet].

[34] Vilagut G, Ferrer M, Rajmil L, Rebollo P, Permanyer-Miralda G, Quintana JM (2005). The Spanish SF-36 health questionnaire: a decade of experience and new developments. Gaceta Sanitaria.

[35] Badia X, Salamero M, Alonso J (2002 ). The measurement of health: guide of measurement scales in Spanish.

[34] Coons SJ, Rao S, Keininger DL, Hays RD (2000). A comparative review of generic quality-of-life instruments. PharmacoEconomics.

[37] Lugo L, García H, Gómez C (2006). Reliability of the SF-36 health quality of life questionnaire in Medellín, Colombia. Revista Facultad Nacional de Salud Pública.

[38] Lugo L, Vera C, García H (2014). Health related quality of life in Medellín and its metropolitan area, with application of SF-36. Revista Facultad Nacional de Salud Pública.

[39] Picavet HSJ, Hoeymans N (2004). Health related quality of life in multiple musculoskeletal diseases: SF-36 and EQ-5D in the DMC3 study. Annals of the Rheumatic Diseases.

[40] Sprangers MAG, de Regt EB, Andries F, Van Agt HME, Bijl RV, de Boer JB (2000). Which chronic conditions are associated with better or poorer quality of life?. Journal of Clinical Epidemiology.

[41] Salaffi F, Sarzi-Puttini P, Girolimetti R, Atzeni F, Gasparini S, Grassi W (2009). Health-related quality of life in fibromyalgia patients: a comparison with rheumatoid arthritis patients and the general population using the SF-36 health survey. Clinical & Experimental Rheumatology.

[42] Birtane M, Uzunca K, Taştekin N, Tuna H (2007). The evaluation of quality of life in fibromyalgia syndrome: a comparison with rheumatoid arthritis by using SF-36 health survey. Clinical Rheumatology.

[43] Belenguer R, Ramos-Casals M, Siso A, Rivera J (2009). Clasificación de la fibromialgia. Revisión sistemática de la literatura. Reumatología Clínica.

[44] Alishiri  GH, Bayat N, Fathi Ashtiani A, Tavallaii SA, Assari S, Moharamzad Y (2008). Logistic regression models for predicting physical and mental health-related quality of life in rheumatoid arthritis patients. Modern Rheumatology.

[45] López-García E, Banegas J, Pérez-Regadera A, Gutiérrez-Fisac J, Alonso J, Rodríguez-Artalejo F (2003). Reference values of the Spanish version of the SF-36 health questionnaire in an adult population aged over 60 years. Medicina Clínica.

